# The taste for health: the role of taste receptors and their ligands in the complex food/health relationship

**DOI:** 10.3389/fnut.2024.1396393

**Published:** 2024-05-30

**Authors:** Gabriella Morini

**Affiliations:** University of Gastronomic Sciences, Pollenzo, Italy

**Keywords:** taste receptors, extra-oral, T2R, metabolism, microbiota, immunity

## Abstract

Taste, food, and health are terms that have since always accompanied the act of eating, but the association was simple: taste serves to classify a food as good or bad and therefore influences food choices, which determine the nutritional status and therefore health. The identification of taste receptors, particularly, the G protein-coupled receptors that mediate sweet, umami, and bitter tastes, in the gastrointestinal tract has assigned them much more relevant tasks, from nutrient sensing and hormone release to microbiota composition and immune response and finally to a rationale for the gut–brain axis. Particularly interesting are bitter taste receptors since most of the times they do not mediate macronutrients (energy). The relevant roles of bitter taste receptors in the gut indicate that they could become new drug targets and their ligands new medications or components in nutraceutical formulations. Traditional knowledge from different cultures reported that bitterness intensity was an indicator for distinguishing plants used as food from those used as medicine, and many non-cultivated plants were used to control glucose level and treat diabetes, modulate hunger, and heal gastrointestinal disorders caused by pathogens and parasites. This concept represents a means for the scientific integration of ancient wisdom with advanced medicine, constituting a possible boost for more sustainable food and functional food innovation and design.

## Introduction

The “food is medicine” concept and approach has been rooted in humans, as proven by the first cookbook published in print around 1,470 by Bartolomeo Platina, “De honesta voluptate et valetudine” (“On right pleasure and good health”) (1). It is actually not just a cookbook but a systematic treatment of culinary arts, dietetics, warnings about the nature of foods, and their nutritional and healing power, with indications and contraindications of different items and preparations and much more that authors of previous centuries had illustrated on the matter. The crucial role of food and food consumption patterns on health have been confirmed by scientific research, from earlier to contemporary times, with new timely discoveries that go hand in hand with an increasingly complex vision (2). If the initial studies concerned a few nutrients and their balance and were addressed to all people, currently there are available databases like FooDB that record the presence of more than 70,000 distinct biochemicals in food (3). Molecular and personalized nutrition are attempting to unravel every organism’s responses to nutrients at a molecular level. Moreover, in the recent decades, diet has been recognized as one of the key factors for the selection and development of trillions of microorganisms that constitute our microbiota, and growing evidence suggests that the relationship between an individual and its microbiota may underlie broad effects of diet on human health and disease (4).

The involvement of taste in this scenario has been, for a long time, limited to its participation in the control of motivational processes that guide eating behavior and food choices, allowing the recognition of nutrients, while avoiding potential toxic compounds (5, 6). The identification of taste receptors, particularly the G protein-coupled receptors (GPCRs) that mediate sweet, umami, and bitter tastes, in the gastrointestinal (GI) tract [as well as in many other locations, not the subject of this article, (7)] has revealed many more relevant tasks for them, from nutrient sensing and consequent hormone release to microbiota composition and immune response (8–10).

## Taste receptors

In humans, taste is mediated in the buccal cavity by two types of specific transmembrane receptors grafted at the apex of the taste receptor cells (TRCs): ion channels, which mediate sour and salty tastes, and GPCRs, for sweet, umami, and bitter tastes (11). The epithelial sodium channel ENaC is a possible receptor for salty taste (sensitive to Na^+^), but still there is no consensus as to whether it is the only one (12). Quite recently, there has been an agreement on the proton-selective ion channel Otop1 as the detector of acidic stimuli (13), as well as of alkali and ions able to modify the pH (14, 15).

Sweetness and umaminess are sensed mainly by two heterodimeric receptors: taste receptor type 1 member 2 (T1R2) and member 3 (T1R3) for the sweet taste receptor, while T1R1 and T1R3 constitute the principal umami receptor (16). T1Rs present a long extracellular N-terminal domain, the so-called Venus flytrap module (VFM), forming the main binding site of the receptor (for sugars, aspartame and sucralose among sweet compounds, and for glutamate and nucleotides among umami ones). The recognition of other tastants and taste modulators occurs in different domains, like the seven transmembrane domain (TMD) and the cysteine-rich domain (CRS) (17–19).

More articulated is the system to perceive bitter taste. To date, over 1,300 bitter compounds have been isolated and tested on human bitter receptors [bitterDB, (20)], approximately 250 of which are of natural origin (21), but it is estimated that there may be up to tens of thousands of them. For a comparison, the known sweet compounds are in the order of the hundreds [SweetenersDB; (22)]. For this reason, vertebrates are equipped with a great number of bitter taste receptors, and their number correlates with the fraction of plants in their diet (23, 24). In humans, 26 active receptors dedicated to bitter compounds, taste receptor type 2 (T2Rs), have been identified, one of them being active in some populations only (25, 26). Some of these receptors are relatively “specialized,” i.e., capable of responding to a limited number of bitter compounds, others, called “promiscuous,” are generalist, i.e., capable of recognizing a larger number of even very chemically different compounds (24). However, others are “monogamous” and hyper-specialized, recognizing only one class of compounds. To date, two are still “orphans” T2Rs, which means that they are not activated by any bitter compound tested so far. T2Rs are more conserved in structure than in sequence, and thus, they are not easy to classify, although they are often considered class A GPCRs, presenting a short N-terminal extracellular domain (25). Moreover, some compounds act as agonists for certain T2Rs and as antagonists for others (27), making structure–bitterness relationship studies almost non-feasible. When comparing bitter taste receptors from different species, for most of them, no one-to-one orthologs are identified as they are organized in species-specific gene expansion groups (28, 29), and therefore, it is not straightforward to draw conclusions about their functionality in humans from animal studies. In addition, the interpretation of bitter taste as a means of rejecting potentially toxic compounds is certainly too simplistic since bitter compounds are not necessarily toxic, nor all toxic compounds taste bitter (30). Indeed, many bitter compounds, which include phenolics, terpenoids, alkaloids, and glucosinolates, have proven to have beneficial health effects (31, 32), and diets including higher amounts of bitter-tasting plants or phytochemicals have been associated with better health (33, 34).

## Taste receptors and nutrient sensing in the GI tract

Most of the nutrients perceived as taste stimuli (particularly the macronutrients) are also under homeostatic control, and therefore, their introduction has to be detected to start and regulate their uptake. Not surprisingly, the GI tract is the largest hormone-producing organ in the body, with enteroendocrine cells (EECs) representing only 1% of the intestinal epithelium, yet capable of producing more than 20 hormones (35). These cells, scattered from the stomach to the rectum, make direct contact with what is present in the GI tract—food (both nutrient and non-nutrient compounds), its digestion products and residues, molecules produced by the microbiota, etc.—and release gut hormonal responses able to convey nutrient-related information to the brain, where they are integrated with other endocrine and neural inputs, generating responses that ultimately control feeding behavior and energy homeostasis through efferent outputs (gut–brain axis) (36).

Among the sensors that account for the ECCs capability to respond to the variety of nutrient stimuli, sweet umami and bitter taste receptors have been identified (37–39): upon activation, these taste receptors trigger the release, among others, of cholecystokinin (CCK) and glucagon-like peptide-1 (GLP-1). CCK has a central effect via the vagal afferents, slowing gastric emptying and therefore reducing food intake, and a GI effect, increasing pancreatic enzymes secretion and gallbladder contraction (35). GLP-1 has its target in pancreatic β-cells, indicating a directly promoted insulin secretion (and inhibition of glucagon) with a relevant effect on glucose control. GLP-1 is the main hormone with “incretin effect” (the increased insulin response after oral ingestion of glucose, compared to that observed when the same glucose load is administered parenterally). With the incretin effect accounting for 50–70% of insulin secreted in response to oral glucose, GLP-1 and its analogs became an effective therapeutic strategy to treat type 2 diabetes (35, 40). GLP-1 receptors are also expressed in the ganglion of the vagus nerve, suggesting a potential gut–vagus–pancreatic islet neural loop. Its release is also followed by slower gastric emptying.

While the contribution of sweet and umami receptors to the regulation of glucose blood level and feeding control could be intuitive since they respond to nutrients, it is surprising that this effect is also mediated by bitter taste receptors due to the fact that, most of the times, the ligands of these receptors are not macronutrients and, therefore, do not provide energy to the body.

## The metabolic roles of bitter taste receptors

Over the centuries, various bitter medicinal plants (most of them non-cultivated) have been used in traditional medicines and bitterness intensity as a food/medicine watershed (41). In particular, for plants used in different folk medicines worldwide to treat diabetes, the bitter connection is obvious. In recent years, for many of them, the glucose control activity has been explained at the molecular level through bitter taste receptor activation by some of their components and subsequent GLP-1 release (42–47).

The alkaloid berberine, produced by several plants growing in different continents and used in traditional medicines to treat diabetes (in the midst of other diseases), has been proven to release GLP-1 (also) through bitter taste receptor pathways (48). Bitter melon (*Momordica charantia L*.) is a food/medicine widely consumed throughout sub-tropical countries (China, India, Thailand, East Africa, and Central and South America), particularly in the management of diabetes. It is a hallmark of Okinawa diet, with goya champuru being Okinawa’s signature dish. It is very likely that its activity in glycemic control is due to several mechanisms, with the release of GLP-1 via bitter taste receptor activation being one of them (49). In the context of glycemic control via nutrition, particular interest should be given to steviosides, secondary metabolites sharing the common aglycone steviol (ent-13-hydroxykaur-16-en-18-oic acid) but different in the number and types of sugar residues, extracted from the leaves of the *Stevia rebaudiana Bertoni*. This plant is native to Paraguay, where it has a long history of use for its sweetness and to treat several diseases, with diabetes being one of the most relevant (50). Currently, steviosides are widely used as intensive sweeteners and still the only ones of natural origin allowed in Europe. A recent study (51) demonstrated that their activity on glucose control is due to GLP-1 release triggered by the activation of bitter taste receptors (T2R4 in particular) while excluding any participation by sweet taste receptors in the process. Another reason why steviosides are of particular interest is that they enhance pancreatic β-cell function by potentiation of TRPM5 channel activity (52). As TRPM5 is also expressed in EEC cells, the activity of steviosides as modulators of GLP-1 also through this route has not been excluded (51).

Another specific example of traditional use of plants in metabolic control, explained at molecular level via a bitter taste receptor expressed in the gut (T2R14), is the anti-hunger activity of *Hoodia gordonii*, an indigenous plant of South Africa, Botswana, and Namibia (53).

Bile acids (BAs) are produced in the liver during the catabolism of cholesterol and then conjugated with glycine or taurine to make them amphipathic and therefore able to work as emulsifiers, exerting a key role in intestinal absorption and transport of dietary lipids. Through multiple and complex pathways, only partially unraveled, they have a role also in incretin secretion, glucose and energy metabolism, endoplasmic reticulum stress, and GI microbiota composition (55). The activation of several human bitter taste receptors by physiological concentrations of bile acids has been proven (54), and it has been hypothesized that this activation might contribute to the role of BA in glucose control (55, 54). Moreover, this finding indicates the role of bitter taste receptors as chemosensors for endogenous ligands, thus opening new fields of discoveries.

The activation of T2Rs and their involvement in digestion and satiety has also been shown at gastric level. Caffeine, which is able to activate five bitter taste receptors in humans, induces gastric acid secretion, known to have a role in satiation, as well as an important signal to initiate gastric protein digestion (56). Interestingly, a similar effect was proven for bitter amino acids (57) and peptides produced during the gastric digestion of caseins (58), demonstrating the participation of bitter taste receptors in the well-known satiety properties of proteins and amino acids.

## Bitter taste receptors in innate immunity and in shaping the microbiota

One of the first, and apparently less logical to explain, location in which extra-oral bitter receptors were identified were the airways. In fact, while their expression in the digestive apparatus can still be inherent to food and its components, this is not at all the case for the respiratory tract. However, since the first evidences of their presence in solitary chemosensory cells in the nasal epithelium it was first brilliantly intuited and then proven their function to respond to acyl-177 homoserine lactones (AHLs) produced by Gram-negative bacteria as quorum-sensing signals (59). This intuition was absolutely endorsed by the following studies, proving that bitter taste receptor activation by AHLs triggers the release of nitric oxide (NO), a toxic defense molecule against pathogens, and increases ciliary beating (60). Similar cells in the gut, tuft cells, detect parasite and bacterial homeostatic dysregulation and initiate a type II immune response, therefore having a role in microbiota composition and, finally, in maintaining gut barrier integrity (61). It has been demonstrated that they monitor intestinal contents using succinate (the main microorganisms and helminths parasite metabolite) as well as sweet and bitter taste receptors (62, 63). In particular, bitter taste receptors activation has been related with their role in repairing gut barrier integrity compromised by obesity and dysbiosis (64).

The effect on T2R38 bitter taste receptor-mediated immunity of the bitter agonist allyl isothiocyanate (AITC), one of the most abundant phytochemicals in plants of the genus Brassica (produced from the precursors glucosinolates in a degradation reaction catalyzed by myrosinase), has also been proven at the systemic level using human peripheral blood, demonstrating that a single intake of an AITC-containing food product resulted in its concentration at plasma levels able to trigger bitter taste receptor-dependent immune cell responses (65).

With microbiota emerging as one of the key components in health status and in the genesis of many diseases, including cancer (in particular colorectal cancer), the modulation of bitter receptors in the GI could also be exploited to develop targeted microbial therapies or as chemopreventive agents though the diet, to promote overall health and against tumorigenesis (66, 67).

To underline their role in monitoring endogenous compounds and microbiota metabolites, it is noteworthy that bitter taste receptors are the only chemosensory receptors from intronless genes, usually associated with rapidly changing expression levels in response to stress (68).

## Conclusion

Taste receptors, given their broad extra-oral expression, have relevant and extensive chemoreception roles, and are much more than simply gatekeepers of food ingestion and data providers for perception. For this reason, some authors even suggested addressing them with a different name rather than simply “taste receptors” (68, 69). Indeed, the discovery of extra-oral taste receptors and their functions gave them the role of ecological sensors in inter-kingdom communication, working at the interface between us and the outside world and also between us and our inner world. In particular, bitter taste receptors in the GI tract, which have been proven to be involved in metabolic responses to food molecules, their metabolites, and endogenous compounds, as well as in immunological response and in microbiota composition, became an important factor in shaping the health and unraveling the complex diet/health relationship, as well as in the gut–brain axis, emerging as potential new drug targets (and their ligands new medications).

During the revision process of this perspective, a nutraceutical formulation based on *Cinchona* bark, *chicory*, and *Gentian* roots has been presented in an open article after having being patented (70), with indications for appetite and weight management. Interestingly, the combination of plants has been appropriately designed to simultaneously target and stimulate several different T2Rs, proving the validity of the proposed approach in this perspective.

The information about the most advanced knowledge on bitter taste receptors and the function of their ligands should be spread to food producers: the growing demand in dietary proteins, especially plant protein ingredients and their derived final products, coincides with the problem of undesirable sensory properties, among which bitterness is often reported. Attention should be paid to debittering through bitter taste receptor inhibition since this action could affect the described metabolic roles and the microbiota composition. In addition, the knowledge on bitter taste receptors should be spread to consumers so that they can realize, or better to say, regain, the value of taste as a tool of knowledge (a form of intelligence) to identify bioactive compounds in food, and this understanding could contribute to their sensory acceptability.

Finally, due to the recent discoveries and the rooted role of bitterness in the food–medicine continuum in traditional knowledge, bitter taste receptors and their ligands could represent a tool for the scientific integration of ancient wisdom with conventional and advanced medicine, constituting a boost also for sustainable and functional food innovation and design.

## Data availability statement

The original contributions presented in the study are included in the article further, inquiries can be directed to the corresponding author.

## Author contributions

GM: Conceptualization, Methodology, Writing – original draft, Writing – review & editing.
